# A Comprehensive Study on the Biological Activity of Elderberry Extract and Cyanidin 3-*O*-Glucoside and Their Interactions with Membranes and Human Serum Albumin

**DOI:** 10.3390/molecules23102566

**Published:** 2018-10-08

**Authors:** Paulina Strugała, Sabrina Loi, Barbara Bażanów, Piotr Kuropka, Alicja Z. Kucharska, Aleksandra Włoch, Janina Gabrielska

**Affiliations:** 1 Faculty of Life Sciences and Technology, Department of Physics and Biophysics, Wrocław University of Environmental and Life Sciences, C.K. Norwida 25, 50-375 Wrocław, Poland; paulina.strugala@upwr.edu.pl (P.S.); aleksandra.wloch@upwr.edu.pl (A.W.); janina.gabrielska@upwr.edu.pl (J.G.); 2 Department of Biomedical Sciences, University of Cagliari, Cittadella Universitaria S.S. 554, Km 4.5, Monserrato, 09042 Cagliari, Italy; sabryloi@hotmail.it; 3 Faculty of Veterinary Medicine, Department of Pathology, Wrocław University of Environmental and Life Sciences, C.K. Norwida 31, 51-375 Wrocław, Poland; 4 Faculty of Veterinary Medicine, Department of Biostructure and Animal Physiology, Wrocław University of Environmental and Life Sciences, C.K. Norwida 31, 50-375 Wrocław, Poland; piotr.kuropka@upwr.edu.pl; 5 Faculty of Biotechnology and Food Science, Department of Fruit, Vegetable and Plant Nutraceutical Technology, Wrocław University of Environmental and Life Sciences, J. Chełmońskiego 37/41, 51-630 Wrocław, Poland; alicja.kucharska@upwr.edu.pl

**Keywords:** elderberry extract, cyanidin 3-*O*-glucoside, lipid membrane, albumin, cytotoxic properties, MCF-7

## Abstract

In our research we used the extract from dietary supplement of elderberry (EE) and its dominant anthocyanin—cyanidin 3-*O*-glucoside (Cy 3-gluc). By interacting with a model membrane that reflects the main lipid composition of tumor membranes, the extract components, including Cy 3-gluc, caused an increase in packing order, mainly in the hydrophilic region of the membrane. It can thus be stated that EE caused a rigidifying effect, which is fundamental for understanding its anticancer and antioxidant activity. This study represents the first attempt to unravel the mechanism of interaction of elderberry extract with membranes. The results of the interaction with human serum albumin (HSA) proved that the studied substance quenches the fluorescence of HSA through a static mechanism in which the main interaction forces are Van der Waals and hydrogen bonding. The antioxidant activity of EE and Cy 3-gluc on liposomal membranes, antiradical properties and ability to inhibited the activity of the enzymes cyclooxygenase-1 and cyclooxygenase-2 were also demonstrated. Moreover, the anticancer activity of EE and Cy 3-gluc on human breast adenocarcinoma cell line were investigated. In addition, EE also exhibited the ability to form lipid aggregates in the form of liposomal capsules that can be applied as carriers of active biological substances, and the highest efficacy of EE encapsulation was obtained for multilayered liposome formulations.

## 1. Introduction

Elderberry has been used in folk medicine as analgesic, antivirus, anti-inflammatory and heart disorders, haemostatic, antitussive, antiparasitic and to soothe burns [1] to treat various illnesses such as stomach ache, sinus congestion, constipation, diarrhea, sore throat, common cold, and rheumatism [2], and also to prevent or delay the onset of chronic age-related diseases [3,4]. Its nutritional and therapeutic values are mainly due to the presence of polyphenol compounds, especially a subgroup, the anthocyanins. In vitro data on the antioxidative, antiviral and possible anti-inflammatory, antibacterial and antiproliferative, effects are very promising, although there are insufficient clinical data and further studies are required [5]. The compounds have met with increasing attention due to a strong body of evidence that supports their bioactivities.

In spite of the large body of studies on the biological activity of flavonoids, including anthocyanins, the mechanism of their activity on the molecular level is not sufficiently known. To a large extent such a mechanism depends on their ability to interact with the biological membrane, which is the first natural barrier on their way into the cell interior [6,7].

A dietary supplement widely available on the world market that contains elderberry extract was used in the study. In view of that, the biological properties of black elderberry in vitro have been well documented in recent years. However, little is known about how it impacts the cell membrane. At the moment, there are no published data about the mechanism of interaction of the extract from elderberry with membranes, in particular membranes that mimic the lipid phase of cancer membranes. Therefore, the proposed research is a first attempt to unravel that mechanism. The interaction with the mimic lipid membrane could trigger and mediate certain biological effects of substances of the anthocyanins group. Moreover, to exert its beneficial effect, extract components need to be transported and distributed into the cells, and this can be affected by binding to human serum albumin. Therefore, the parameters for binding between anthocyanin and other extracts components and HSA must be considered when determining the viability of these compounds in transport into and within the cell [8]. The use of anthocyanin-rich extracts is limited because of the low stability of anthocyanins under the environmental conditions experienced during processing and/or storage [9,10]. What is more, the high instability of isolated anthocyanins from berry-type fruits has a direct impact on their potential health benefits, in fact causing a reduced biological activity and bioavailability. The best way seems to be the microcapsulation technology of anthocyanins that can be used to improve the stability and/or bioavailability these substances [11].

The purpose of this comprehensive study was to explain how the extract from elderberry and cyanidin 3-*O*-glucoside interact with the mimic lipid phase of tumor membranes. For these tests, we used the fluorimetric method and two types of probes. The interaction of these substances with the DPPC liposomal membrane was also observed using the nuclear magnetic resonance (^1^H-NMR) method. Additionally, we studied the effect of those compounds on human breast cancer cell line (MCF-7). To address the problem of molecular interaction of Cy 3-gluc and EE with human albumin, a fluorescence spectroscopy investigation was performed. Finally, we investigated the antioxidant (in liposomal system) and anti-inflammatory activity of studied substances and their micro-encapsulation efficiency in lipid aggregates as a potential drug carrier or diet supplement.

## 2. Results and Discussion

### 2.1. Phenolic Content by HPLC/LC-MS Method

By the HPLC/MS chromatographic method was performed quantitative and qualitative analysis of the elderberry extract. The content of polyphenolic compounds was expressed in mg/g of dry mass preparation (Table 1), it was found to be 160.95 mg per 1 g of preparation. The predominant compounds present in the extract were anthocyanins, that accounted for about 84.70%, and protocatechuic acid (8.70%) of identified compounds. We also detected low amounts of quercetin 3-*O*-glucoside (1.70%), quercetin (0.83%), and two acids from the hydroxybenzoic acid group (1.80%). In the group of anthocyanin compounds cyanidin 3-*O*-glucoside predominates (87.80% of the anthocyanins). Also present are peonidin 3-*O*-glucoside (9.70%), cyanidin 3-sambubioside-5-glucoside (0.54%), and cyanidin 3,5-diglucoside (0.44%).

Other studies on elderberry have shown similar polyphenolic compositions. For example, cyanidin derivatives were dominant in elderberries, in particular cyanidin 3-*O*-glucoside as the main anthocyanin in elderberries [12]. In other studies it was shown that cyanidin *O*-sambubioside is the main compound in elderberry extract [13,14,15]. Kšonžekowá et al. [16] determined the total anthocyanin content in extract of elderberry as equal to 483.7 mg/g of DM, while other literature data indicate a 8.33–101.40 mg/g of DW range [17], which are either much higher or lower than the value determined in the present work (136.96 mg/g of DW). To a large degree it depends on the method of extraction and type of reagents used. The content and composition of anthocyanins vary between different species, cultivars and the date of fruit harvesting. Factors such as light, temperature and stress during cultivation significantly modify their synthesis and accumulation [18,19].

### 2.2. Packing Order and Fluidity of Membrane

Efficacy of the biological activity of phenolic compounds (e.g., antioxidant and antitumor activity) depends on e.g., the way the substances interact with biological membranes that may result in structural changes within them. To date, relatively modest studies have shown that polyphenols modify the properties of membranes, e.g., when acting against tumors.

We have analyzed the effect exerted by elderberry extract components on the structural properties of a lipid mimic membrane. That kind of study, to the best of our knowledge, has not been reported previously. Here, by using the fluorimetric method with two fluorescent probes that become embedded at different depths of the membrane bilayer we have determined the effect of elderberry extract on properties of the hydrophobic and hydrophilic area of the MM.

The effect of elderberry extract on the hydrophilic area of the mimic membrane was studied with the use of MC540 probe, which is a dye that locates at or near the membrane interface, slightly above the domain of the glycerol backbone lipids [20,21]. Our results show that components of EE at the concentration studied (2–20 µg/mL) caused a significant decrease in the fluorescence intensity of the MC540 probe compared to control (Table 2). The probe’s fluorescence reflects the packing order of lipids in the bilayer—the weaker the fluorescence the more ordered are the lipids of the polar part of membrane [22]. The results suggest that studied substances caused a significant increase in packing order of polar heads of membrane lipids. In an earlier study we observed a similar effect. The main component of elderberry, cyanidin 3-*O*-glucoside, in the range 4–20 µM, induced a marked decrease in MC540 fluorescence intensity in the range of approx. 4.6–17.6% relative to control [20]. Moreover, in our earlier work a similar effect was found for extracts from black currant and chokeberry, which in the range 3–15 µg/mL induced a marked decrease in MC540 fluorescence intensity when anchored in egg phosphatidylcholine [23].

The PNA marker is a hydrophobic molecule that becomes incorporated near the ester-carbonyl region of the acyl chains of lipids [24]. The measured change in probe anisotropy in the hydrophobic region of membrane caused by the penetrating substance results in a change in its microenvironment, which is interpreted as change in membrane microfluidity [25]. Our results showed that EE modifies the properties of the hydrophobic region of MM membrane to a lesser degree than in the hydrophilic region, where the probe MC540 is embedded. With 15 and 20 µg/mL concentrations the extract components induced ca. 4.5% increase in anisotropy compared to control but the changes observed are at the error level (Table 2). Increase in isotropy signifies that extract molecules caused a decrease in fluidity in the area of hydrocarbon chains of membrane lipids.

Literature reports indicate the existence of a strict correlation between the fluidity of tumor membranes and the extent of cancerous changes within the membrane [26,27,28]. For instance, it was shown that fluidity of leukemia membranes is two times greater than fluidity of normal cells [29], and this relation holds true also for other tumors [30,31,32]. In the light of these reports and our research, it is legitimate to state that structural changes in MM induced by cyanidins from elderberry are one of the key mechanisms of potential antitumor activity. Our results are in accord with results of other authors, e.g., Tsuchiya showed that flavonoids (e.g., myricetin, quercetin, kaempferol, apigenin) at 10 µM caused reduced membrane fluidity of mimetic membranes that reflects the composition of membrane lipids of cancer [33]. In addition, as a consequence of those results one can suggest that extract components took position mainly in the surface, hydrophilic region of the membrane. Such distribution of the antioxidative molecules on the surface of membrane may constitute a kind of barrier against attacking free radicals. We postulate that it is a way of the antioxidative action of polyphenolic compounds.

### 2.3. ^1^H-NMR Measurements

**Figure 1:**
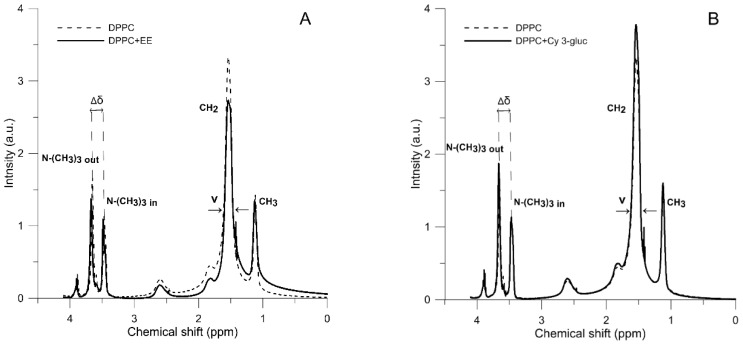
^1^H-NMR spectra of liposomes formed from pure DPPC liposomes and DPPC with (**A**) elderberry (EE, *v*/*v* 400:1), (**B**) Cyanidin 3-*O*-glucoside (Cy 3-gluc, mol/mol 100:1). PrCl_3_ (4 mM) added to the samples before measurement.

Results obtained with the fluorimetric method have been augmented with measurements made using the ^1^H-NMR method. Figure 1 presents the ^1^H-NMR spectra of DPPC liposomes and DPPC liposomes with addition of elderberry extract (400:1 *v*/*v*) (Figure 1A) and with addition of 1 mol % cyanidin 3-*O*-glucoside (Figure 1B). Several bands are visible in the spectra, that correspond to the major molecular features of the DPPC membranes: -CH_3_ and -CH_2_ groups of the hydrophobic regions of the membrane as well as the bands of -N^+^-(CH_3_)_3_ from the polar head region of the membrane. Presence of praseodymium ions in the liposome medium results in the split of the ^1^H-NMR band (Δδ) corresponding to the ammonium group, -N^+^-(CH_3_)_3_, owing to the pseudo-contact shifts produced by the shift reagents from the group of lanthanides (e.g., Pr^3+^), [34]. The ratio of the areas under the signal assigned to the outer layer (I_Out_) to that assigned to the inner layer (I_In_) is proportional to the number of choline heads in the outer and inner layers. It is obvious that the ratio of the two signals greater than 1 relates to unilamellar liposomes.

Addition of EE or its component Cy 3-gluc caused the following changes in the spectra parameters (Table 3): in the full width at half height (ν) of the ^1^H-NMR bands, in the ratio I_Out_/I_In_ and in the splitting (Δδ) of the band corresponding to the ammonium group -N^+^-(CH_3_)_3_, from the outer and inner leaflets of the membrane. Addition of EE also caused a change in the full width at a half height (ν) of the all main ^1^H-NMR bands. Small increases in the case of -(N^+^-CH_3_)_3 Out_ (1.7%) and -CH_2_ groups (4.6%) were observed, whereas for groups -N^+^-(CH_3_)_3 In_ (11.9%) and -CH_3_ (43%) pronounced increases were noted. The presence of Cy 3-gluc caused an increase in ν by 4% for outer leaflet of the membrane and only slight increase for inner leaflet of the membrane. Moreover, presence of EE changed the I_Out_/I_In_ ratio, from 1.1545 in pure DPPC to 1.2221 in liposomes with addition of examined extract (approx. 6%) (while Cy 3-gluc increased this value more significantly from 1.1545 to 1.322 (approx. 15%). Addition of EE and Cy 3-gluc also resulted in an increase in the splitting parameter of the resonance maximum (Δδ) corresponding to polar head group, the increase was approx. 3% and 8%, respectively. The finding from this investigation is that EE components, including Cy 3-gluc, become incorporated into DPPC membrane and exert influence, probably during the formation of hydrogen bonds, on the spectral parameters of formed liposome membrane. This means that intercalated molecules induced restriction in mobility of molecular segments of the DPPC lipid membrane, affecting the local order.

### 2.4. Antioxidant Activity and Free-Radical Scavenging Activity

So far, there is little knowledge on the antioxidant activity of elderberries relative to membranes, which are the first target for free radicals from external sources that attack cells at the organism level.

**Figure 2:**
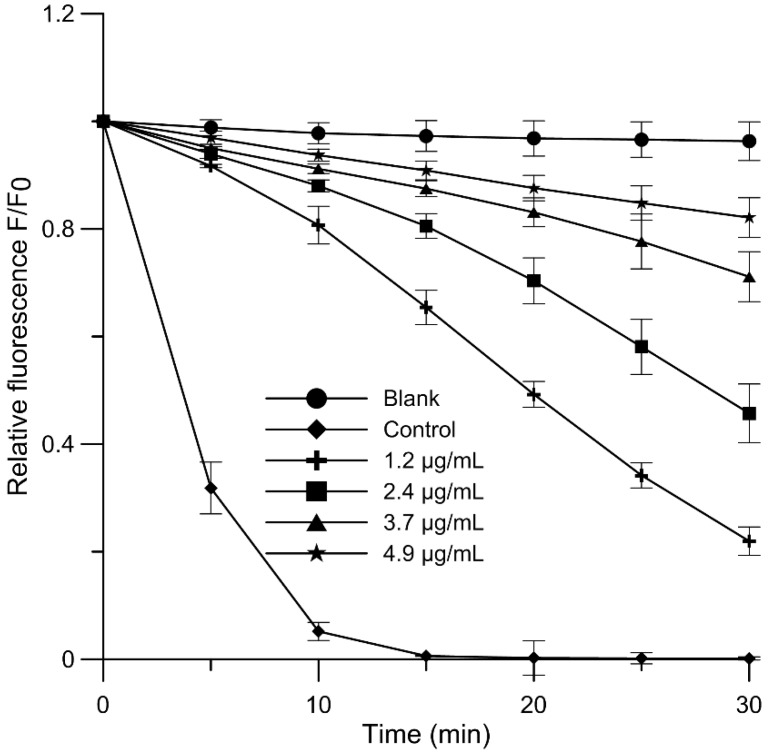
Relative fluorescence intensity of DPH-PA probe as a function of oxidation time of MM membranes for AAPH radical in the presence of elderberry extract at chosen concentrations. The relative changes in fluorescence intensity *F*/*F*_0_ are a measure of the degree of lipid peroxidation, (*F*_0_—fluorescence in the control sample, *F*—fluorescence of samples in the presence of study molecules).

The investigation of the antioxidant activity of elderberry extract was conducted here on a MM membrane. The results of antioxidant activity of studied EE and Cy 3-gluc are shown as relations between relative intensity of DPH-PA fluorescence incorporated to liposomes in the presence of test substances and time of oxidation. Relative fluorescence intensity kinetic curve of the probe DPH-PA in the presence of elderberry extract for mimic liposomes is shown in Figure 2, and for Cy 3-gluc it was published in the previous article [20]. With increasing EE concentration the intensity of fluorescence increases in proportion to the degree of lipid membrane oxidation. Based on the plot of oxidation kinetics (also for Cy 3-gluc), the percentage of oxidation inhibition after 30 min was calculated for EE and Cy 3-gluc. Next, the percent of oxidation inhibition versus concentration of the antioxidant was plotted and from it the IC_50_ parameter was estimated. These IC_50_ values, listed in Table 4, indicated that EE had 72% smaller capacity to inhibit oxidation of lipids than Cy 3-gluc, whereas both EE and Cy 3-gluc protected the lipid membrane against peroxidation induced by AAPH to significantly greater degree than ascorbic acid (IC_50_ = 22.80 ± 2.19 µg/mL) when we used phosphatidylcholine liposomes [35]. It can be stated that the tested substances were from 8.9 to 31.7 times better antioxidants than ascorbic acid. Moreover, it turns out that elderberry extract, whose content of identified phenolic compounds is similar to that of blackcurrant, shows a 2.7 fold higher antioxidant activity than blackcurrant [23,36].

We also confirmed antiradical capacity of EE and Cy 3-gluc using a DPPH^•^ test, determining the EC_50_^DPPH^ parameter, results being compiled in Table 4. The parameter (in µg/mL) assumed the values 3.54 ± 0.16 and 4.25 ± 0.84 for EE and Cy 3-gluc, respectively. In comparison with the activity of ascorbic acid for neutralizing the DPPH^•^ free radical which is EC_50_^DPPH^ = 3.26 ± 0.001 µg/mL [35], EE and Cy 3-gluc are not significantly less effective scavengers of this radical in red-ox reactions. It can be judged from the analysis of the values compiled in Table 4 that the ability of EE and cyanidin to protect the mimetic lipid membrane against peroxidation is greater than their effectiveness in leveling off the DPPH^•^ free radical. It is believed that in addition to the high redox potential of an antioxidant molecule, an effective protection of MM membranes against free radical attack requires adequate and strategic location of extract molecules in the membrane. On the basis of obtained values of IC_50_ and contents of individual compounds in the extracts it can be stated that antioxidant activity of these compounds against membrane oxidation is largely determined by the physicochemical properties of Cy 3-gluc. Antioxidant activity of elderberry extracts has been also confirmed in the current literature. The studies performed by Kšonžeková et al. [16] confirmed the higher antioxidant status of elderberry extract, which is rich in cyanidin glycosides, compared to the activity of blueberry or bilberry extracts, and additionally indicated no cytotoxic effect on porcine intestinal epithelial cells. Studies by Bratu et al. [37] inform, for example, that *Sambucus nigra* powder fruit extract in vitro has a very high antioxidant activity (quantified by a photoluminescence method), and no mutagenic effect at concentration below 0.1 g/dL, which makes it recommendable for application in food industry. The review of Sidor and Michałowska [15] shows both a large variety of methods for making black elderberry extracts as well as the results of different tests of antioxidant activity.

### 2.5. Cyclooxygenase Activity

The results for inhibition of enzymes involved in the inflammatory reactions of the body are expressed by the IC_50_ parameter (in µg/mL), as shown in Table 4. They show that EE is about 29% more efficient in COX-2 inhibition (IC_50_ = 46.58 ± 5.22) than COX-1 (IC_50_ = 65.26 ± 4.53). IC_50_ values for Cy 3-gluc are 5- and 6-times lower than those for EE (500 and 450% for COX-1 and COX-2, respectively). This means that Cy 3-gluc is potentially a very effective substance that inhibits proinflammatory processes that depend on COX enzyme activity. Furthermore, its potential anti-inflammatory activity is higher than that reported in vitro by indomethacin (approx. 25% and 5%, respectively, compared with the corresponding COX-1 and COX-2 parameters for indomethacin) [23]. Fruit and flowers of elderberry have a long tradition in herbal medicine of being used to reduce inflammation and in treatment of colds and flu [15]. Literature reports confirm the anti-inflammatory activity of elderberry. For example it was found that elderberry extract stimulated the production of proinflammatory cytokines (IL-1β, IL-6, IL-8) as well as anti-inflammatory cytokine (IL-10) [38]. Moreover, in Farrell et al. [39] it was shown that elderberry extract feeding would attenuate the low-grade inflammation and insulin resistance in the mouse model of obesity.

### 2.6. Microcapsulation of Elderberry

Although anthocyanin encapsulation technology is a difficult task, it can be a very good tool for improving the stability and/or bioavailability of anthocyanins [40,41,42]. From literature reviews it follows that methods/techniques of anthocyanins encapsulation with potential application for the food industry and for clinical (therapeutical) use are under intensive development. Fang and Bhandari [40] have reviewed polyphenol encapsulation techniques and considered spray- and freeze-drying processes for encapsulating anthocyanins using maltodextrin as wall material. Excipients used for encapsulating various substances have included maltodextrin, β-cyclodextrin, pullulan, glucan gel, curdlan, sodium alginate and pectin [43]. In addition, others have used pectin and shellac to encapsulate anthocyanins [44]. In this study we decided to use only one natural building material for making microcapsules, which is soy lecithin. Capsulation with soy lecithin liposomes ensures the formation of non-toxic for the organism microcapsules as potential drug carriers [45].

The objective of the present study was to develop and characterize different methods (systems) of encapsulation, which would be most effective for anthocyanin-rich elderberry extract. In addition, we wanted to examine which of the methods for formation of lecithin microcapsules (liposomes) proves to be most effective. The EE was incorporated in liposomes produced by three formulation methods: vigorous shaking (LMV), sonication (SV) and extrusion (E-SUV). Liposomes/vesicles formed by these methods differ in size and the number of layers. They can be LMV—large multi-lamellar, SV—small, E-SUV—extrusion small unilamellar vesicles. Results for the encapsulation efficiency of EE in liposomes and sizes of the vesicles are compared in Table 5. By dynamic light scattering (DLS) and zeta potential measurement we characterized stability of the liposome dispersions. The highest efficiency of EE encapsulation in soy PC liposomes was obtained for LMV liposomes (36.4 and 43.0% for both EE concentrations used) and smallest for SV and E-SUV in the presence of 1.25 mg/mL of EE (about 13%). It can be seen that the size of liposomes strongly depends on the mode of their formation and in case of LMV and SV their diameter much increased with increasing concentrations of EE. It is interesting that in the presence of 0.5 mg/mL EE by extrusion through 100 nm filter smaller homogeneous vesicles (93 ± 5 nm, Polydispersity index PdI = 0.109 ± 0.049) are formed than those formed without EE (132 ± 6 nm, PdI = 0.041 ± 0.028). Literature reports say that encapsulation effectivity of hydrophilic substances in liposomes (compared with lipophilic ones) is relatively low. Nii and Ishii [46] determined the effectiveness of enclosing the medicine 5-fluorouracil in MLV liposomes from egg lecithin as equal to ca. 12% for hydrogenated purified egg yolk lecithin while it was ca. 17%, whereas the lipophilic medicine ibuprofen was encapsulated with ca. 90% effectiveness. In Bryła et al. [47] the effectiveness of elderberry capsulation in liposomes formed by a freeze and thaw method with soybean lecithin was 25%. This value compares well with our result (23.5%) for E-SUV via pressing through a polycarbon filter. The physical parameters of the obtained liposomes can bring about higher homogeneity of the nanoparticles, compared with those obtained by Bryła et al. [47]. For comparison, the nanocapsulation efficacy in Vit C was 47.16% for small liposomes (73.9 nm) formed of soybean lecithin [48], being thus close to that obtained in this work for LMV (43.0%, 9732 nm in diameter and EE: Lipid ratio 1.25:20 *w*/*w*).

### 2.7. Fluorescence Quenching of Human Serum Albumin

Albumin, as the main protein of blood plasma, plays many important roles in the human body. It has natural affinity to many exo- and endogenic substances. Studies concerning the influence of biologically active compounds on protein constituents of the human blood circulation system (mainly serum albumin) are of great interest, because the interaction of potential drugs with blood components can affect both their bioavailability and the functioning of biomolecules. Determination of the degree of binding between a biologically active substance, as a potential medicine, and albumin seems to be the basic factor that decides about its health beneficial properties [49].

**Figure 3:**
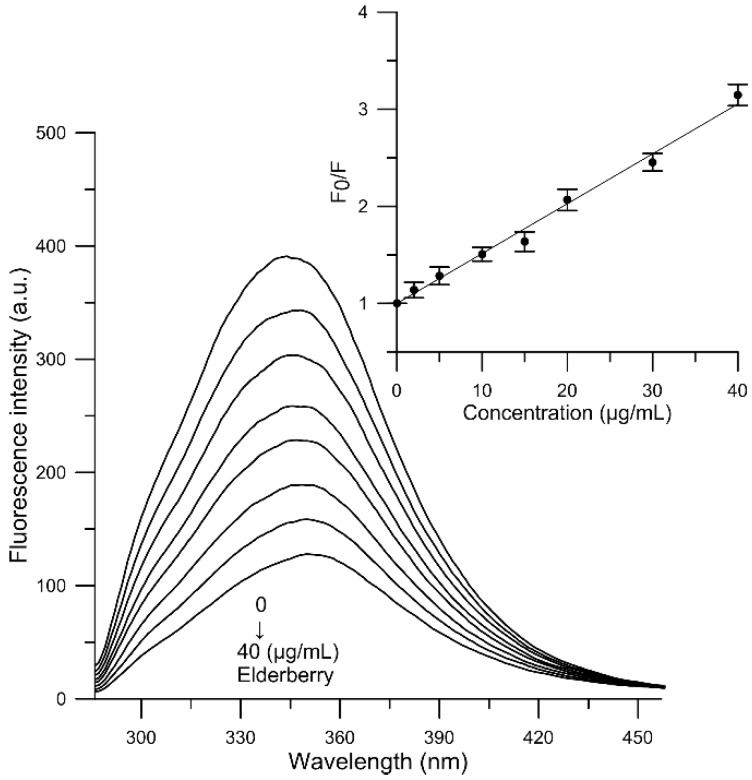
Emission spectra of HSA in the presence of various concentrations of elderberry extract and Stern-Volmer plots of *F*_0_/*F* against concentration of study molecules (HSA = 1.5 × 10^−5^ M, λ_ex_ = 280 nm, T = 310 K).

The fluorescence emission spectra of human serum albumin in the absence and presence of elderberry extract were measured at the four temperatures 300, 305, 310 and 315 K. The effect of EE extract on fluorescence intensity of HSA at 305 K is shown in Figure 3. Addition of elderberry extracts (2–40 µg/mL) to HSA causes a prominent fluorescence quenching when the concentration of EE is increased. The fluorescence intensity of HSA in the absence of elderberry extract at the emission maximum was about 389 a.u. (Figure 3, line 1), and after addition of 40 µg/mL extract the fluorescence intensity dropped to 124 a.u. (Figure 3, the last line). The decrease in fluorescence intensity was about 68%. This result suggests that elderberry extract is able to interact with HSA and quench its intrinsic fluorescence. These results are also in accord with our previous reports. Decrease in fluorescence intensity upon addition of the main anthocyanin of elderberry extract confirms the binding of Cy 3-gluc to HSA [20].

Results on the natural fluorescence quenching of HSA as a function of temperature were further analyzed using the Stern-Volmer equation [50], to explain the mechanism of fluorescence quenching:

(1) $$\frac{F_{0}}{F}=1+K_{q}\tau_{0}\left[Q\right]=1+K_{SV}\left[Q\right]$$

where *F*_0_ and *F* refer to fluorescence intensities of HSA prior to and after addition of the quencher (EE), *K_q_* is a bimolecular quenching constant, *τ*_0_ is the lifetime of fluorophore in the absence of quencher (5 × 10^−9^ s) [51], [*Q*] is the concentration of quencher, and *K_sv_* is the Stern-Volmer quenching constant (*K_sv_* = *K_q_·τ*_0_).

Based on Equation (1), the binding constant *K_SV_* for the complex EE-albumin was calculated using linear regression of the plot of *F*_0_/*F* versus [*Q*]. The plot of *F*_0_/*F* against concentration of EE at 310 K is shown in Figure 3. The plots were linear in the 2–40 μg/mL range of the components of EE concentration. Fluorescence quenching can be accounted for by two interaction mechanisms, known as dynamic or static quenching. Dynamic quenching, which is caused by collisions and higher temperatures, results in larger diffusion coefficients. In static quenching increased temperature is likely to destabilize the bound complexes, and thus lower values of the static quenching constants are expected [52].

As evidenced by our results (Table 6), the *K_sv_* of EE decreased with increasing temperature, which indicated that the mechanism of the system EE-HSA was all static quenching. Our earlier studies [20] have shown that *K_SV_* for Cy 3-gluc decreased with increasing temperature and the values of the quenching rate constant (*K_q_* = 2.804 × 10^12^ M^−1^·s^−1^, e.g., at 310 K) were all far greater than the limiting diffusion constant of the biomacromolecule (1.0 × 10^10^ M^−1^·s^−1^) [49]. Taking into consideration the above experimental results and our earlier studies, it can be concluded that the HSA fluorescence quenching may originate from the formation of a complex between Cy 3-gluc present in elderberry extract and albumin via a static quenching process.

In addition, the quenching process was analyzed through the modified Stern-Volmer plot to get information about binding constant (*K_b_*) and number of binding sites (*n*) by implementing the following equation:

(2) $$\log[(F_{0}-F)/F]=\log K_{b}+n\log\left[Q\right]$$

Both the values *K_b_* and *n* were obtained by plotting $\log[(F_{0}-F)/F]$ versus log[*Q*]. Binding parameters at different temperatures are presented in Table 6. The results show that the values of *K_b_* decrease with temperature increase, which is ascribed to the fact that the force involved in the binding process (the complex of EE with HSA) must have been weakened with increased temperature [53]. It is also worth noting that the values of *n* at the experimental temperatures were almost on the same level, that is 1, suggesting that only the existence of a single binding site in HSA for components of EE could be detected (Table 6).

The molecular forces contributing to the interactions of proteins with molecules include hydrogen bonding, Van der Waals, electrostatic and hydrophobic interactions. In order to elucidate the type of interaction in the complex formation process, after calculating thermodynamic parameters of the binding process by using well known van’t Hoff’s equation [34,54,55] we calculated the enthalpy change (*ΔH*), free enthalpy change (*ΔG*) and entropy change (*ΔS*) in the binding process, the results being presented in Table 6. The bond between HSA and EE results in negative values of *ΔG*, *ΔS* and *ΔH*. The negative values for free enthalpy change (*ΔG*), which were from −10.286 to −8.360 kJ/g·mL^−1^, demonstrate that the interaction process of HSA and EE at these temperatures is spontaneous.

Such a relation was also obtained in our previous work for Cy 3-gluc [20]. When *∆H* < 0 and *∆S* > 0, the electrostatic force dominates in the interaction; when *∆H* < 0 and *∆S* < 0, van der Waals interactions and hydrogen bonds dominate and when *∆H* > 0 and *∆S* > 0, hydrophobic interactions dominate in the binding process [56]. Our results suggest that the Cy 3-gluc compound and EE extract bind to HSA mainly as a result of hydrogen bonds and van der Waals forces.

### 2.8. Cytotoxic Properties on BHK-21 and CEF Cell Lines

The elderberry extract and Cy 3-gluc were evaluated for their cytotoxic activities on the BHK-21 and chicken embryo fibroblasts (CEF, normal) cell lines. A cytotoxic effect was observed after 24 h incubation with different concentrations of tested compounds. In the case of EE the cytotoxic effect was observed in a concentration higher than 1:20, since concentration 50 µg/mL was regarded as not toxic. Analogously, the substance Cy 3-gluc was nontoxic at a dilution of 1:80 (25 µg/mL).

**Figure 4:**
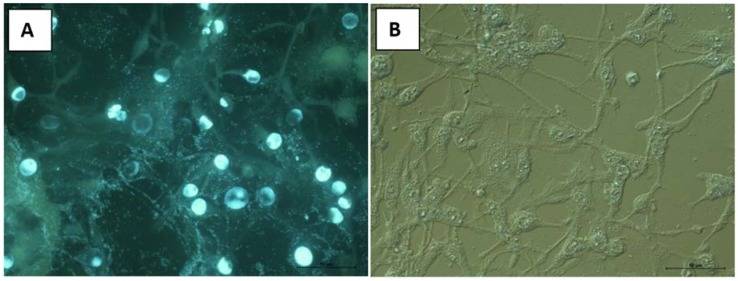
BHK-21 cell line stained with Hoechst after administration of substance EE at 50 µg/mL (**A**). No changes in this cell line, BHK cell line in Nomarski contrast. Control group (**B**).

For instance, Figure 4 presents the effect of extract on BHK-21 cell line by using a fluorescence microscope. The BHK-21 cell culture is characterized by the presence of elongated, spindle-like cells with long protrusions connected to other cells. The nucleus, located in the center of the cell, is surrounded by abundant cytoplasm. Large, round or oval nuclei contain one or more nucleoli, and the cytoplasm contains numerous rough materials that can be visualized by the contrast-phase technique. The fluorochromes reveal a moderate number of divisions in this cell line. The mitochondrial activity remains at normal level. The results show that after administration of EE at 50 µg/mL (Figure 4B) no significant changes were observed.

**Figure 5:**
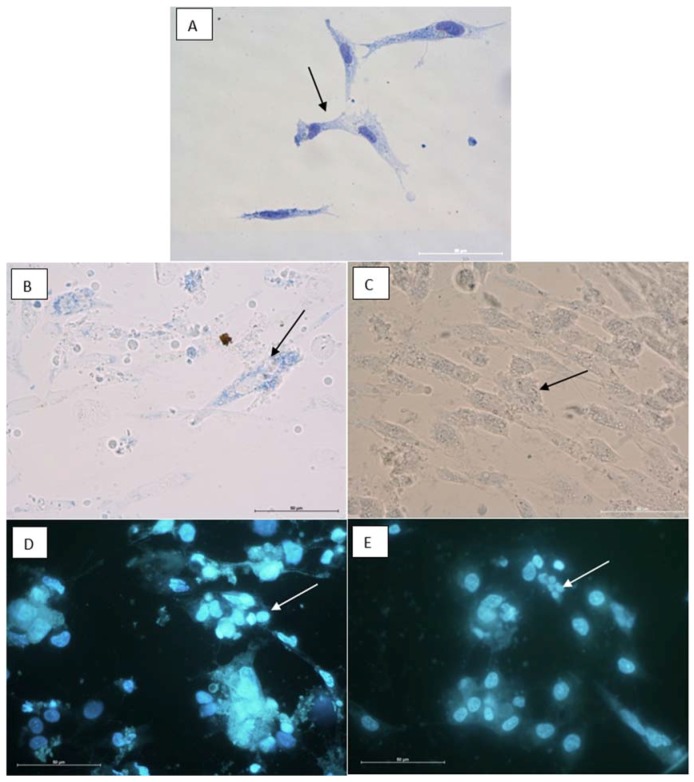
Changes in fibroblasts 45 min after administration of elderberry extract and cyanidin 3-*O*-glucoside. Control group (**A**), experimental group: EE in concentration 50 µg/mL (**B**,**D**), Cy 3-gluc in concentration 25 µg/mL (**C**,**E**). Note the change in cell shape (arrow). This process was more advanced in Cy 3-glu group than EE. Mitochondria are present in cells as small green/blue granules and their activity remains at the same level (arrow). In experimental group mitochondria are localized mainly around nucleus in contrast to control group, Mag × 200 scale bar represent = 50 μm.

The changes in the CEF cell culture suggest that the substances cause deep changes in fibroblast morphology, however do not alter the activity of mitochondria. After administration of substances the cells start to produce numerous follicles, shrink and became apoptotic. During this entire process, the mitochondria remain intact. The analysis of the influence of EE and Cy 3-gluc on this cell line revealed increased apoptosis, more frequent in the Cy 3-gluc group. The cells became round and contain numerous follicles in the cytoplasm (Figure 5).

### 2.9. Antitumor Properties on MCF-7 (Human Breast Adenocarcinoma Cell Line)

The antitumor activity of the extract and Cy 3-gluc was evaluated in vitro using the MCF-7 cell line by the fluorescence method. Observation of cell morphology is the most direct way to know whether the cells are undergoing apoptosis, so we used DAPI fluorescent dye that binds strongly to DNA and stains both live and fixed cells [57]. Our results showed that the MCF-7 cell culture is characterized by the presence of elongated, spindle like cells with long processes connected to other cells. Cells are present as aggregates in the center or as single cells in peripheral regions. Large, round or oval nuclei contain 1 or more nucleoli, and cytoplasm contains numerous rough material that can be visualized by the contrast phase technique.

**Figure 6:**
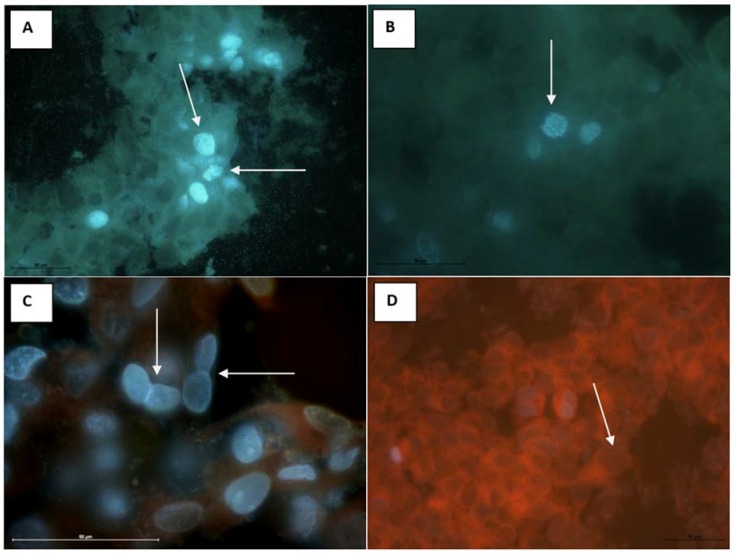
Numerous dividing cell (arrow) in control group, DAPI, 600 × magnification, scale bar = 50 μm (**A**,**B**). Inhibited cytokinesis in experimental group (MCF-7), concentration EE is 50 µg/mL (**C**). Visible cells containing two nuclei (arrow) surrounded by cytoplasm containing cytoskeleton (red) Hoechst and Rhodamine Mag × 1000, scale bar represent = 50μm. Inhibited cytokinesis in Cy 3-gluc group concentration 25 µg/mL (**D**). Inhibited cytokonesis. In all cells two or more nuclei are noted (arrow) Rhodamine B, Mag × 400.

The fluorochromes revealed numerous figures of cell division, indicating a fast growth of the tumor cells (Figure 6). The experimental groups showed similar levels of cytostatic effects of EE and Cy 3-gluc regardless of their concentration Figure 6C,D. The reduction in cell division number was clearly observed in all examined groups. Some of the cells were inhibited at S-phase or telophase. In many cells inhibition of cytokinesis was observed. This deregulation of cell cycle have impact on cell morphology. The cells changed the shape and most of them became smaller, round with short processes, aggregated and attached to each other, containing 2 nuclei with reduced cytoskeleton structure. Therefore clusters of round cells with two or more nuclei were prominent in examined material. Single, degraded cells were observed in every tested group, but compared to the control group (Figure 6A,B) it was not caused by substance tested.

Our results are in agreement with previous data showing the anticancer effects of anthocyanins and anthocyanin-rich extracts on breast cancer cells. For example, Olsson et al. [58] reported that extracts from blackcurrant, grape and bilberry at 5 mg/mL caused approx. 20–45% growth inhibition on MCF-7 cells. In addition, Chen et al. [59] showed that cyanidin 3-*O*-glucoside purified from black rice inhibited human breast cancer cell HS578T growth. A significant characteristic of cancer cells is their uncontrolled cell cycle, that leads to continuous division and proliferation [60,61]. Studies have shown that anthocyanins can selectively inhibit the proliferation of cancer cells but have little influence on the proliferation of normal cells [62,63]. In Thole et al. [64] it was shown that both cultivated *Sambucus* *nigra* and wild *Sambucus* *canadensis* fruits demonstrated significant chemopreventive potential through strong induction of quinone reductase and inhibition of cyclooxygenase-2, which is indicative of anti-initiation and anti-promotion properties, respectively. Fractions of *S. canadensis* extract showed inhibition of ornithine decarboxylase, an enzyme marker related to the promotion stage of carcinogenesis. 

## 3. Materials and Methods

### 3.1. Materials

Cholesterol, DPPH^•^, indomethacin, *N*,*N*,*N*′,*N*′-tetramethyl-*p*-phenylenediamine (TMPD), arachidonic acid from porcine liver, cyclooxygenase 1 from sheep, cyclooxygenase 2 human recombinant, 2,2′-azobis(2-amidinopropane) dihydrochloride (AAPH), human serum albumin (lyophilized powder, essentially fatty acid free) were purchased from Sigma–Aldrich (St. Louis, MO, USA). The probes 3-[*p*-(6-phenyl)-1,3,5-hexatrienyl]propionic acid (DPH-PA), Merocyanine 540 (MC540), *N*-phenyl-1-naphthylamine (PNA) were purchased from Molecular Probes (Eugene, OR, USA). 1-Palmitoyl-2-oleoyl phosphatidylcholine (POPC), 1-palmitoyl-2-oleoyl phosphatidylethanolamine (POPE) and 1-stearoyl-2-oleoylphosphatidylserine (SOPS) were obtained from Avanti Polar Lipids (Delfzijl, The Netherlands). Cyanidin 3-*O*-glucoside was obtained from Extrasynthese (Genay, France) and soya been lecithin (SPC) from Phospholipid GmbH (Köln, Germany).

### 3.2. Elderberry Supplement

A dietary supplement widely available on the world market, that contains fruit elderberry extract *Sambucus williamsii* Hance (country of origin USA, batch number CH2512021) was used in the study. The process of obtaining the elderberry extract was described, with small modifications, by Strugała et al. [36]. In brief, powders of elderberry supplement were dissolved in 70% of ethanol-water solution (50 g/200 mL) and incubated for 15 min under ultrasound (Sonica, Rho, Italy), and after that the supernatant was decanted. Such extractions were repeated several times. The resulting supernatant was then evaporated with ethanol and next was passed through a column filled with Amberlite^®^ resin (XAD4). The column was washed with distilled water until the wash-out free of total sugars. Polyphenolic extract of elderberry was obtained after washing the column with 70% ethanol. The collected fraction was evaporated at 40 °C in a vacuum evaporator until dry mass. Extract thus obtained (EE) was stored at a temperature of 4 °C until assayed.

### 3.3. High-Performance Liquid Chromatography/Mass Spectrometry (HPLC/MS) Methods

Quantitative and qualitative analysis of the components present in the elderberry extract was performed by a HPLC/LC-MS chromatographic method [65]. Details of methodology are included in our previous work [65,66]. Flavonols were detected at 360 nm, phenolic acids at 280 nm, and anthocyanins at 520 nm. Flavonols were qualified as quercetin 3-*O*-glucoside, protocatechuic acid asprotocatechuic acid, and anthocyanins as cyanidin 3-*O*-glucoside. The results were calculated as mg of compound in 1 g dry mass of extract (mg/g of DM). All determinations were performed in duplicate.

### 3.4. Liposome Preparation

The study was carried out using liposome membranes with special lipid composition, consisting of the same ingredients as in the membrane of tumor cells (mimic membrane—MM). The procedure presented previously by Strugała et al. [20] was according to Tsuchiya [33], with small modifications. Shortly, a chloroform solution of a lipid mixture containing 48 mol % POPC, 24 mol % POPE, 8 mol % SOPS, and 20 mol % cholesterol dried in vacuum under nitrogen for about 1.5 h was prepared. A phosphate buffer of pH 7.4 was added, and the sample vortexed to obtain a milky suspension of multilamellar vesicles. The final concentration of lipids in the vesicle suspension was 0.1 g/L. Such suspension was then sonicated for 10 min at 0 °C with a 20 kHz sonicator (Sonics, Newtown, CT, USA) The vesicle suspension obtained was used as the source of liposomal membranes having the mimic bilayer structure.

### 3.5. Affinity of Elderberry Extract for Cell-Mimic Membranes

The fluorescence experiments were carried out using different fluorescent probes that embedded in the hydrophilic or hydrophobic region of liposomal MM, according to a procedure described in our earlier works [20,23]. We assumed that study compounds could modify rather the surface layer, and we applied two probes (MC540 and PNA) to monitor the interface of the MM bilayer. The MC540 dye was used according to a method described before by Manrique-Moreno et al. [22]. The fluorescence intensity of MC540 emission was determined and expressed as relative intensity change in relation to the control (*F*_0_/*F* − 1) as a function of EE and Cy 3-gluc concentration in the range 2–20 µg/mL, where: *F*_0_-fluorescence in the absence of compounds, *F*—fluorescence in presence of studied compounds. Packing density in the MM was determined on the basis of anisotropy (A) of PNA calculated according to the formula:

(3) $$A=\frac{I_{∥}-GI_{⊥}}{I_{∥}+2GI_{⊥}}$$

where $I_{∥}$ and $I_{⊥}$ are fluorescence intensities observed in directions parallel and perpendicular to the polarization direction of the exciting wave, respectively. G is an apparatus constant dependent on emission wavelength. Measurements were carried out at 310 K, using a spectrofluorimeter with a built-in polarization attachment (SFM-25, Kontron Instruments, Zürich, Switzerland). The experiment was performed in three independent replicates (*n* = 3).

### 3.6. ^1^H-NMR Measurements

The experiments were carried out according to the procedure described in our earlier work [35]. Mixtures of phospholipids (DPPC) and EE or Cy 3-gluc were co-dissolved in chloroform/ethanol mixture (55:1/*v*:*v*) at the respective concentration. The lipid concentration in the sample was 3.2·10^−1^ M and that of EE 400:1 (*v*/*v*), in the case of Cy 3-gluc it was 3.2·mM. The samples were first evaporated under a stream of nitrogen and then in vacuum (2 h). Then the samples were hydrated with D_2_O and vigorously shaken (approx. 15 min) at a temperature above the main phase transition of the lipid (41 °C). Next the lipid suspension was sonicated for 15 min with a 20 kHz sonicator (VCX-130, Sonics, Newtown, CT, USA) above the main phase transition of lipid, and a homogenous lipid dispersion was obtained. Briefly, before measurements 4 mM praseodymium trichloride (PrCl_3_·6H_2_O) was added to the sample of 0.6 mL liposome suspension. ^1^H-NMR spectra were recorded on an Avance DRX 500 spectrometer (Bruker, Milton, ON, Canada). 500 MHz ^1^H-NMR parameters were as follows: Spectral windows 12,019 Hz, digital resolution 0.183 Hz, acquisition and delay time of 2.73 s and 1.00 s, respectively, acquisition temperature 325 K.

### 3.7. Antioxidant Activity

Antioxidant activities of extract were determined using the fluorimetric method described in our earlier work [35]. The studies were carried out on cell MM in phosphate buffer (pH 7.4) at 0.1 mg/mL that contained the fluorescent probe DPH-PA (1 µM). Use was made of the relationship between DPH-PA fluorescence intensity and concentration of free radicals. The probe’s fluorescence decreased with its rising oxidation caused by free radicals supplied by AAPH of final concentration 1 M at 37 °C. As a measure of the degree of lipid membrane oxidation was assumed the value of relative intensity of DPH-PA fluorescence. It was calculated as a ratio of fluorescence intensity after 30 min of oxidation in the presence of studied compounds (EE and Cy 3-gluc) to the initial value of intensity. The concentration of compounds was changed in the range 1.0–2.9 µg/mL. The measurements were conducted with a fluorimeter (Cary Eclipse, Varian, San Diego, CA, USA). The inhibition of lipid oxidation was calculated on the basis of the following formula:

(4) $$\%\;inhibition=\frac{\left(F_{S}-F_{C}\right)}{\left(F_{B}-F_{C}\right)}\cdot 100\%$$

where *F_S_* is relative fluorescence of the probe oxidized by AAPH in the presence of compounds, *F_C_* is relative fluorescence of control sample oxidized by AAPH without compounds, *F_B_* is relative fluorescence of the blank sample. The experiment was performed in five independent replicates (*n* = 3).

### 3.8. Free-Radical Scavenging Assay

The effect of EE and Cy 3-gluc on reduction of DPPH^•^ radical concentration was measured spectrophotometrically, as previously described by Brand-Williams et al. [67]. The experiment is described in detail by Strugała et al. [36]. Reduction of DPPH^•^ in the sample after 15 min incubation with an antioxidant (of fixed concentration) was determined using the formula:

(5) $$\%\;Reduction=\frac{\Delta A_{0}-\Delta A}{\Delta A_{0}}\times 100\%$$

where: *ΔA*_0_ is a change of absorbance at λ = 517 nm after 15 min in the absence of EE or Cy 3-gluc; and *ΔA* is change in absorbance at λ = 517 nm after 15 min in the presence of EE or Cy 3-gluc. All determinations were performed in five replicates (*n* = 5).

### 3.9. Cyclooxygenase Activity

The anti-inflammatory activity of the EE extract, established on the basis of a modified method given in the work by Jang and Pezzuto [68], was assayed by a spectrophotometric measurement of inhibition of activity of cyclooxygenase COX-1 and COX-2. The experiment is described in detail in our earlier work [36]. In short, the experimental procedure was as follows: to a cuvette containing Tris-HCl buffer (pH 8.0) the following were successively added: EE (or Cy 3-gluc), hematin (0.1026 mM) and a cyclooxygenase (COX-1 and COX-2) at 1 mg/mL. After mixing and incubation (approx. 3 min), TMPD was added at 24.35 mM. To initiate the reaction, arachidonic acid was added at a concentration of 35 mM. The final volume of the sample was 1 mL. Changes in absorbance of the sample were followed for 3 min by measuring it at 1 min intervals, using a spectrophotometer at a wavelength of 611 nm (Cary 100 Bio, Varian, San Diego, CA, USA) in relation to a reference sample. The measurements were carried out at room temperature. The control sample, instead of the extract, contained the right amount of solvent. The experiment was performed in five replicates (*n* = 5).

### 3.10. Effectiveness of Microencapsulation of Elderberry Extract (EE) in Liposomes and Vesicle Size Determination

Microencapsulation of the EE extract was performed with liposomes formed of PC using the methods of mechanical shaking (LMV), sonication (SUV) and extrusion (EUV). The extracts (at concentration 0.5 mg/mL or 1.25 mg/mL) were added while hydrating the lipid film with TRIS-HCl buffer of pH 7.4 at 20 mg/mL of SPC at room temperature. During the hydration the sample was vigorously shaken (15 min) at the room temperature (LMV). In a part of experiments, the preparations of liposomes (LMV) were sonicated for 3 min using a 20 kHz sonicator (SUV) (VCX-130, Sonics, Newtown, CT, USA). The third method of forming liposomes containing EE, was carried out by extrusion through polycarbonate filters. The LUV suspension was extruded 31 times through a 100 nm pore membrane (EUV). After the end of preparing the liposomes, the probes were centrifuged at 30,000 *g* and 4 °C for 2 h in the case of LMV preparation (Thermo Scientific, Darmstadt, Germany) or in the case of SUV and EUV at 100,000 *g* and 4 °C for 2 h in a Beckman ultracentrifuge (Beckman Coulter Optima^TM^ L-90K, Woburn, MA, USA) in order to separate the capsulated EE in liposomes from their free form. The control samples containing only a suspension of lipid vesicles were obtained in the same ways as liposomes with encapsulated EE. The concentration of EE closed in liposomes separated from supernatant was determined photometrically at λ = 570 nm (Cary 100 Bio, Varian, San Diego, CA, USA). Standard linear calibration curves were made in the range 1–450 μg/mL and on this basis the extract concentration in liposomes was calculated (y = 0.3996x + 0.0529; y = absorbance; x = EE concentration, R^2^ = 0,983). The percentage of EE entrapment (encapsulation effectiveness) was calculated on the basis of the formula:

(6) $$E\left[\%\right]=\frac{C_{e}\left[\mu g/\mathrm{mL}\right]}{C_{t}\left[\mu g/\mathrm{mL}\right]}\cdot 100\%$$

where: $C_{e}$—concentration of EE entrapped in liposomes, $C_{t}$—initial total concentration of EE. All determinations were performed for three independent preparations (*n* = 3) using a Cary 300 Varian spectrophotometer.

The mean dimeter of the liposomes was determined (multimodal analysis, volume weighted) using a Zetasizer Nano ZS90 (Malvern Instruments Ltd., Malvern, UK).

### 3.11. Fluorescence Quenching of Human Serum Albumin

Analysis of the potential interaction of study compounds with human serum albumin (HSA) was performed according to the work [34], with minor modifications. Fluorescence measurements were performed on a fluorimeter (Cary Eclipse, Varian, San Diego, CA, USA) equipped with 1.0 cm quartz cells and a thermostat bath. All quenching experiments were performed at 300, 305, 310 and 315 K for HSA in a phosphate buffer solution of pH 7.4 of final concentration 1.5 × 10^−5^ M. The excitation wavelength was set at 280 nm and the emission spectra were read at 282–460 nm. The excitation and emission slits were both set to 5 nm. Our method consisted in tracking the quenching of natural HSA fluorescence caused by all compounds added successively. The final concentrations varied in the range 2–40 µg/mL. The experiment was performed in three independent replicates (*n* = 3).

### 3.12. Biological Studies

#### 3.12.1. Cytotoxic Properties as Tested on BHK-21and RK-13 Cell Lines

The cell lines RK-13 (rabbit kidney, p. 270, ATCC, Manassas, VA, USA, No CCL-37 TM) and BHK-21 (baby hamster kidney, p.17 ATCC, No CCL-10 TM) incubated in 96-well polystyrene plates were used in this project. EE and Cy 3-gluc were tested using EN 14675 as described earlier [69]. Product test solutions were prepared in Minimum Essential Medium (MEM) supplemented with additional 2% Fetal Bovine Serum (FBS) and L-glutamine. Serial dilutions of tested complexes (dilution step 1:10 from starting concentration of EE 10 mg/mL and Cy-3-gluc 2 mg/mL) were prepared and transferred (50 μL) into cell culture units (wells of microtitre plates) containing suspended in MEM cells (50 μL). Eight units were inoculated with each dilution. Plates were incubated in 37 °C/5% CO_2_ and observed daily for up to 4 days for the development of cytotoxic effect, using an inverted microscope (Olympus Corp., Hamburg Germany; Axio Observer, Carl Zeiss MicroImaging GmbH, Berlin, Germany).

#### 3.12.2. Cytological Investigations as Tested on BHK-21and RK-13 Cell Lines and CEF Cell Cultures

BHK-21 and RK-13 cell lines and the primary cell culture CEF (chicken embryo fibroblasts; 5000 cells per 1 µL) were incubated in Leighon tubes. Primary cell culture was prepared as described previously [70]. After 24 h of incubation MEM was removed and EE and Cy 3-gluc at starting concentrations in volume 0.5 mL were added after 1 h and 24 h. Then the slides was washed in PBS and stained using buffered 1% acridine orange for cytotoxic analysis and with 1% Janus green for mitochondria activity analysis.

#### 3.12.3. Antitumor Properties on MCF-7 (Human Breast Adenocarcinoma Cell Line)

The cell line MCF-7 (ATCC, No HTB-22 TM) incubated in 96-well polystyrene plates and in Leighon tubes were used in this project. Product test solutions were prepared in MEM supplemented with additional 2% FBS and L-glutamine. Serial dilutions of tested complexes (dilution step 1:10 from starting concentration of EE (10 mg/mL) and Cy 3-gluc (2 mg/mL) were prepared and transferred (50 μL) into cell culture units (wells of microtitre plates) containing suspended in MEM cells (50 μL). Eight units were inoculated with each dilution. Plates were incubated in 37 °C/5% CO_2_ and observed daily for up to 4 days for the development of cytotoxic effect, using an inverted microscope (Olympus Corp; Axio Observer, Carl Zeiss MicroImaging GmbH). MCF-7 cells in Leighton tubes were inoculated by EE and Cy 3-gluc substances in starting concentrations in volume 0.5 mL and stained after 1 h and 24 h post inoculation of EE and Cy 3-gluc for 5 min using Rhodamine B and Hoehst 33342. Then the slides were washed in PBS and observed under an Eclipse 80i fluorescence microscope (Nikon, Amsterdam, Netherlands) equipped with a UV-2A filter. The morphometry of a number of dividing cells and cells with inhibited cytokinesis was performed with use of Nis-Elements Ar software (Nikon, Amsterdam, Netherlands). Statistical analysis was performed with use of Statistica 12.0 (StatSoft Polska, Kraków, Poland).

### 3.13. Statistical Analysis

Data are shown as mean values ± standard deviation (SD). The results were analyzed by one-way ANOVA followed by Duncan test. *p* values < 0.05 were considered statistically significant. The program Statistica 12.0 was used for all statistical calculations.

## 4. Conclusions

The in vitro bioactivity of anthocyanins (anticancer and antioxidant) and the fact that membranes are the primary target of biological activity of those compounds has led us to investigate the molecular interaction of Cy 3-gluc and elderberry extract with the lipid membrane that mimics the lipid phase of cancer membranes. We have undertaken such a comprehensive study to find out how the extract from elderberry and cyanidin 3-*O*-glucoside interact with the lipid membrane and human albumin. The Cy 3-gluc compound and other components of EE when incorporated into the polar head group region of membrane caused restriction in motional freedom of the molecules in the hydrophilic part of the membrane bilayer. The ordering effect in the mimic membrane and DPPC membrane was found using the fluorometric and ^1^H-NMR methods. There are data that provided evidence that anthocyanins (and probably other flavonoids) operate as protective agents against fluidization of cancer membrane. In addition, we explored the possibility of EE and Cy 3-gluc affecting albumin—the main protein of blood plasma, expected to be helpful for understanding the mechanism of drug transport within organisms. In accordance with other results obtained, i.e., the high antioxidant capacity demonstrated in this work, we conclude that elderberry extract and its main component Cy 3-gluc exert beneficial effects on cells via intercalation into and interaction with lipid membranes. On the other hand, incorporation into the surface of membrane and the rigidifying effect helps to protect the membranes modified by anthocyanins against oxidative stress induced not only by free AAPH radicals, but also potentially, e.g., by UV irradiation. Moreover, the anticancer activity of EE and Cy 3-gluc on a human breast cancer cell line has been confirmed. Finally, the anti-inflammatory activity (in relation to COX-1 and COX-2) as well as the good effectiveness of closing the elderberry extract in liposomes indicate a potential usefulness for its therapeutic application.

## Figures and Tables

**Table 1:** Content (mg/g) and characterization of phenolic compounds of the elderberry extract preparation.

Phenolic Compounds	Content (mg/g)	R_t_ (min)	[MS] (*m*/*z*)	Ion Mode	MS/MS Fragments (*m*/*z*)
Cyanidin 3-sambubioside-5-glucoside	0.744	4.242	743	+	287
Cyanidin 3,5-diglucoside	0.602	7.08	611	+	287
Cyanidin 3-*O*-glucoside	120.230	7.93	449	+	287
Cyanidin 3-*O*-rutinoside	1.465	9.13	595	+	287
Peonidin 3-*O*-glucoside	13.263	10.27	463	+	301
Quercetin 3-*O*-glucoside	2.703	13.07	463	−	301
Quercetin	1.334	20.80	301	−	
Protocatechuic acid	13.966	2.73	153	−	109
Hydroxybenzoic acid derivative	0.293	6.12	not identified	−	
Hydroxybenzoic acid derivative	0.307	9.52	not identified	-	
**Total**	**160.95**				

**Table 2:** Changes in fluorescence intensity (for MC540 probe) and fluorescence anisotropy (for PNA probe) in the presence of elderberry extract in lipid mimic membranes.

Elderberry Extract/Concentration (µg/mL)	MC540 Intensity Change from Control (%)	PNA Anisotropy Change from Control (%)
2	−5.55 ± 1.96 *	0.015 ± 2.2
5	−16.70 ± 1.88 *	1.38 ± 3.97
10	−30.93 ± 2.64 *	3.77 ± 2.57
15	−40.95 ± 0.16 *	4.53 ± 3.03
20	−45.58 ± 3.12 *	4.41 ± 0.68

Negative values indicate a decrease in fluorescence intensity compared to the control, while positive values indicate an increase in fluorescence intensity/anisotropy compared to the control. Means labelled with an asterisk (*) are significantly (*p* < 0.05) different from control.

**Table 3:** Parameters of ^1^H-NMR spectra at temperature 325 K of DPPC liposomes and DPPC liposomes with addition of elderberry extract (EE, (*v*/*v* 400:1), and DPPC liposomes with addition of cyanidin 3-*O*-glucoside (Cy 3-gluc, mol/mol 100:1).

Liposome Composition	Parameter					
	ν-N^+^-(CH_3_)_3 Out_ (ppm)	ν-N^+^-(CH_3_)_3 In_ (ppm)	Δδ (ppm)	ν-CH_2_ (ppm)	ν-CH_3_ (ppm)	I_Out_/I_In_
DPPC	0.05222	0.03545	0.1810	0.11679	0.05212	1.1545
DPPC + EE	0.05312	0.03966	0.1870	0.12218	0.07459	1.2221
DPPC + Cy 3-gluc	0.05438	0.03568	0.1950	0.11674	0.05621	1.3222

**Table 4:** Antioxidant (IC_50_), antiradical (EC_50_^DPPH^) and anti-inflammatory (IC_50_^COX-1^, IC_50_^COX-2^) parameters for elderberry extract and cyanidin 3-*O*-glucoside and values of IC_50_^COX-1^ and IC_50_^COX-2^, i.e., concentrations at which 50% inhibition of the activity of COX-1and COX-2 occurs.

Parameters/Compound	Elderberry (µg/mL)	Cyanidin 3-*O*-glucoside (µg/mL)
IC_50_^AAPH^	2.57 ± 0.31	0.72 ± 0.09
EC_50_^DPPH^	3.54 ± 0.16	4.25 ± 0.84
IC_50_^COX-1^	65.26 ± 4.53	6.86 ± 0.35
IC_50_^COX-2^	46.58 ± 5.22	7.21 ± 0.28

**Table 5:** Percentage of elderberry extract (EE) encapsulation in soy phosphatidylcholine liposomes (PC = 20 mg/mL) for different methods of liposome formation and mean diameters of liposomes.

Sample	Encapsulation Efficiency (%)	Mean Diameter (nm)
**Liposomes (LMV)**	**-**	4500 ± 227
LMV + EE (0.5 mg/mL)	36.4 ± 1.2	5120 ± 1167
LMV + EE (1.25 mg/mL)	43.0 ± 3.0	9732 ± 718
**Liposomes (SV)**	**-**	181 ± 44
SV + EE (0.5 mg/mL)	16.4 ± 1.0	398 ± 44
SV + EE (1.25 mg/mL)	13.3 ± 1.6	2150 ± 289
**E-SUV**	**-**	132 ± 6
E-SUV + EE (0.5 mg/mL)	23.5 ± 0.7	93 ± 5
E-SUV + EE (1.25 mg/mL)	13.5 ± 0.7	7761 ± 167

Elderberry was in concentrations of 0.5 and 1.25 mg/mL. LMV—large multi-lamellar vesicles, SV—small vesicles, E-SUV—extruded small unilamellar vesicles.

**Table 6:** Quenching (*K_sv_*) and binding (*K_b_*) constants and thermodynamic parameters (*n*, *ΔG*, *ΔH* and *ΔS*) for elderberry extract and human serum albumin at different temperatures. Standard deviations (mean value of three independent experiments) were lower than 10%.

Compound	*T* (K)	*K_sv_* (mL/g)	*K_b_* (mL/g)	*n*	*∆G* (kJ/g·mL^−1^)	*∆H* (kJ/g mL^−1^)	*∆S* (J/(g mL^−1^·K))
Elderberry	300	59.709 × 10^3^	13.273 × 10^3^	0.858	−10.286	−47.494	−123.189
	305	58.558 × 10^3^	10.365 × 10^3^	0.846	−10.183		
	310	51.353 × 10^3^	5.137 × 10^3^	0.770	−9.564		
	315	49.311 × 10^3^	1.557 × 10^3^	0.644	−8.360		
